# Knowledge of human papillomavirus vaccination: A multi-institution, cross-sectional study of allopathic and osteopathic medical students

**DOI:** 10.1371/journal.pone.0280287

**Published:** 2023-01-11

**Authors:** Samuel R. Bunting, Samantha Morris, Julia Chael, Brian A. Feinstein, Aniruddha Hazra, Sarah S. Garber

**Affiliations:** 1 Department of Psychiatry and Behavioral Neuroscience, The University of Chicago Medicine, Chicago, Illinois, United States of America; 2 Pritzker School of Medicine, The University of Chicago Medicine, Chicago, Illinois, United States of America; 3 Department of Psychology, College of Health Professions, Rosalind Franklin University of Medicine and Science, North Chicago, Illinois, United States of America; 4 Department of Medicine, Section of Infectious Diseases and Global Health, The University of Chicago Medicine, Chicago, Illinois, United States of America; 5 Department of Pharmaceutical Sciences, College of Pharmacy, Rosalind Franklin University of Medicine and Science, North Chicago, Illinois, United States of America; The University of Jordan, JORDAN

## Abstract

Human papillomavirus (HPV) vaccination is a well-established and successful tool for preventing HPV-related cancers. However, vaccine uptake remains low, influenced by patient hesitancy around safety concerns and little opportunity to discuss the vaccine with trusted healthcare providers. We conducted a national, cross-sectional study of allopathic and osteopathic medical students regarding knowledge of HPV vaccination guidelines March-April 2021. Analysis sought to identify gaps in knowledge as well as demographic and academic correlates of knowledge. A total of 718 students participated (response rate = 50.8%). While 92.8% of participants identified the connection between HPV and cervical cancer, lower percentages associated HPV with vaginal/vulvar (67.7%), anal (63.3%), and penile (53.9%) cancers. Low percentages of participants correctly identified age of HPV vaccine eligibility (33.3%) and how many doses are needed for full protection (48.1%). This study identifies specific knowledge gaps in medical students’ training on HPV-related cancers and HPV vaccination guidelines. Through addressing these gaps, we may improve HPV vaccine uptake and decrease the incidence of HPV-related cancers.

## Introduction

Each year in the United States (U.S.), an estimated 35,900 people are diagnosed with a Human Papillomavirus (HPV)-related cancer [1]. An estimated 90% of these cases could be prevented by HPV vaccination [1]. However, a 2020 U.S. nationally representative survey found that only 75% of adolescents aged 13–17 received at least 1 dose of the HPV vaccine, only 59% were fully vaccinated, and vaccination rates were lower among male compared to female adolescents [2]. Furthermore, from 2015 to 2018, there was a 79% increase in the percentage of parents who cited safety concerns as a primary reason to refuse initiation of the HPV vaccine for their adolescents [3]. In the general population, HPV knowledge is relatively limited, particularly surrounding its association with cancer as upwards of 77% of people were unaware of the links between HPV and anal, penile, and oral cancers [4].

Improving HPV vaccination rates is a U.S. public health priority. Previous studies have found that physicians may hesitate to mention HPV vaccinations out of fear that patients will refuse the vaccine as well as inadequate time during visits to sufficiently explain the benefits of vaccination [5]. However, evidence suggests that when physicians do take the time to discuss the merits of the HPV vaccine, they may positively encourage their patients to receive the vaccine [6,7]. Among 18–26 year olds, trust in the clinician and clinician recommendation were major factors influencing vaccination decisions [6]. In a study of college students, clinician recommendation to receive the HPV vaccine resulted in participants being nearly five times more likely to be vaccinated [7].

Patient eligibility for the HPV vaccine has expanded since U.S. Food and Drug Administration (FDA) approval in 2006. While the vaccine was initially aimed at adolescent and pre-teen girls, recommendation has expanded to now include both male and female patients, starting as young as age 9 and extending through age 45. Current guidelines recommend patients aged 11–12 receive 2 doses and patients aged 15–26 receive 3 vaccine doses [1]. Patients aged 27–45 are advised to engage in shared decision making with their providers to determine whether vaccination against HPV is appropriate based on individual risk factors and values.

While the link between HPV and cervical cancer is widely-known, HPV is also responsible for oropharyngeal, anal, penile, vulvar, and vaginal cancers, which account for over two-thirds of annual HPV-related cancers [1]. Previous studies of medical students have found that many, 59.7% of students, were not aware of the connection between HPV and these other cancers [8,9]. Given the power of clinician recommendation and knowledgeable explanation of the risks and benefits of HPV vaccination, medical education about HPV vaccination is imperative to ensure vaccination rates meet the public health need.

Previous studies have found knowledge of HPV and HPV vaccination increased as medical students progressed through training [8,10]. Medical students who had personally received the HPV vaccine also reported significantly greater willingness to vaccinate adolescents before ages 15–16, as well as greater willingness to discuss vaccination during visits [11]. However, much of this research was conducted prior to expansion of indications for HPV-vaccination beyond adolescent and pre-teen girls. Continued investigation is needed to assess current medical students’ knowledge of HPV vaccination with the ultimate goals of continuing to increase HPV vaccination rates and decrease the incidence of HPV-related cancers.

Furthermore, most of the previous research on medical students’ HPV vaccine knowledge was conducted with allopathic medical students. However, osteopathic and allopathic medical students differ in their medical school curricula and clinical experiences. Both types of students learn scientific, evidence-based practices and treatments at four-year medical training programs that are examined by the same state licensing boards (but using different exams). That said, osteopathic medical practice centers holistic medicine and musculoskeletal manipulative medicine techniques, while allopathic medicine focuses primarily on symptom alleviation and disease treatment through pharmaceutical interventions [12]. Osteopathic medical education places a specific emphasis on preventive medicine and health promotion, such as vaccinations.

The goals of the current study were to address the following, specific research questions: 1) What are the specific knowledge gaps among medical students regarding U.S. Center for Disease Control (CDC) HPV vaccinations guidelines?; 2) What are the demographic and academic correlates of knowledge of HPV vaccination guidelines?; and 3) Are there specific knowledge deficits that remain among medical students in their final year of training?

## Methods

### Study population and procedure

The study participants were recruited from a cross-sectional sample of students from 16 U.S. allopathic (10) and osteopathic (6) medical schools, which represented a convenience sample of institutions selected for regional diversity. School administrators disseminated study information to potential participants between March–April 2021. Study information sent to students deliberately avoided any mention of HPV to reduce the risk of selection bias. Inclusion criteria for this study were: 1) 18 years of age or older, and 2) currently enrolled in a U.S. allopathic or osteopathic medical school. Interested students who met both inclusion criteria were sent a follow-up email with instructions to access the study. The survey was completed online using QualtricsXM (Provo, UT). Upon completion of the study, participants were sent a debrief message and a $10.00 gift card to an online retailer as compensation.

### Instrument development

The study instrument was developed specifically for use in this study. We developed items related to knowledge of HPV informed by the guidelines and recommendations for HPV immunization as developed by the U.S. Preventive Services Task Force (USPSTF) [1,13]. Items were reviewed by an infectious disease physician with content expertise for accuracy. A focus group of 10 medical students from a single, Midwestern allopathic medical education program completed the instrument and provided feedback on item wording and study administration. We incorporated this feedback prior to distribution of the instrument to the larger sample.

The first section of the study presented students with a list of cancer diagnoses (e.g., cervical, colon, vaginal/vulvar) and asked participants to indicate which cancers could be caused by HPV. The next section presented a series of multiple-choice questions and participants were instructed to select the single best answer for each. Multiple choice questions were developed based on review of the CDC and USPSTF guidelines for HPV-vaccination [1,13]. Multiple choice questions are listed in Table 2 and the complete study instrument is attached as a S1 File.

### Statistical analysis

We calculated descriptive statistics to describe the sample as well as all items included on the knowledge assessment. Next, we calculated the percentage of respondents who correctly answered each item. The percentage who answered correctly was compared between the group of participants in the pre-clinical phase (years 1/2) and clinical-phase (years 3/4) of medical training utilizing Pearson’s chi-squared tests (χ^2^). The total number of correct items was summed and divided by the total number of items to create a percentage of correct knowledge for each participant. The knowledge percentage was compared between demographic and training variables utilizing a one-way analysis of variance (ANOVA) for independent variables with three or more levels and independent samples *t*-tests for type of training, which only had two levels. Multivariable analyses were completed using analysis of covariance (ANCOVA) adjusting for all demographic and training variables. Data management and analysis was conducted using IBM SPSS v27 (IBM Corp., Armonk, NY). The pre-determined level of significance was a *p*-value < 0.05. This study was reviewed and approved by the Institutional Review Board of Rosalind Franklin University (protocol: COP-20-256) on November 2, 2020 and informed consent was obtained from all participants before beginning the study via the online study instrument.

## Results

A total of 1,592 students indicated interest in participating in this study. Of these interested students, 808 completed the study (response rate = 50.8%). We removed 90 due to incomplete responses. This left a final analytic sample of 718.

### Demographics

The largest proportion of the participants in this study were in their first year of training (*n* = 204, 28.4%) and approximately half were in allopathic medical education programs (*n* = 382, 53.2%). Over half of participants were White (*n* = 416, 57.9%), most were heterosexual (*n* = 604, 84.1%), and approximately two-thirds were cisgender women (*n* = 450, 62.7%). Regionally, the greatest number of participants were training in the Midwestern U.S. (*n* = 361, 50.3%). Full demographic information is provided in Table 1. The mean age of participants was 26.3 years (*SD* = 3.25). Comparison of demographics between allopathic and osteopathic student groups is presented in S1 Table. We also compared demographics of the sample responding to the survey to the demographics of medical students nationally to ensure representation without identifying major discrepancies (S2 Table).

**Table 1:** Sample demographics (*N* = 718).

**Year of Training**	***n***	**%**
1^st^	204	28.4
2^nd^	189	26.3
3^rd^	157	21.9
4^th^+	168	23.4
**Type of Training**		
Medicine (allopathic-MD)	382	53.2
Medicine (osteopathic-DO)	336	46.8
**Race/Ethnicity** *		
White	416	57.9
Black	27	3.8
Hispanic/Latino	47	6.5
Asian	239	33.3
Other Race	33	4.6
**Sexual Orientation** *		
Heterosexual (straight)	604	84.1
Homosexual (gay/lesbian)	40	5.6
Bisexual	53	7.4
Different Sexual Orientation	19	2.6
Did Not Answer	2	0.2
**Gender Identity**		
Man (cisgender male)	262	36.5
Woman (cisgender female)	450	62.7
Different Gender Identity	6	0.8
**Region**		
South	70	9.7
Northeast	138	19.2
West	149	20.8
Midwest	361	50.3

*Some totals exceed 718 because participants could select multiple response options for a given demographic characteristic.

### Specific cancer diagnoses

**Fig 1:**
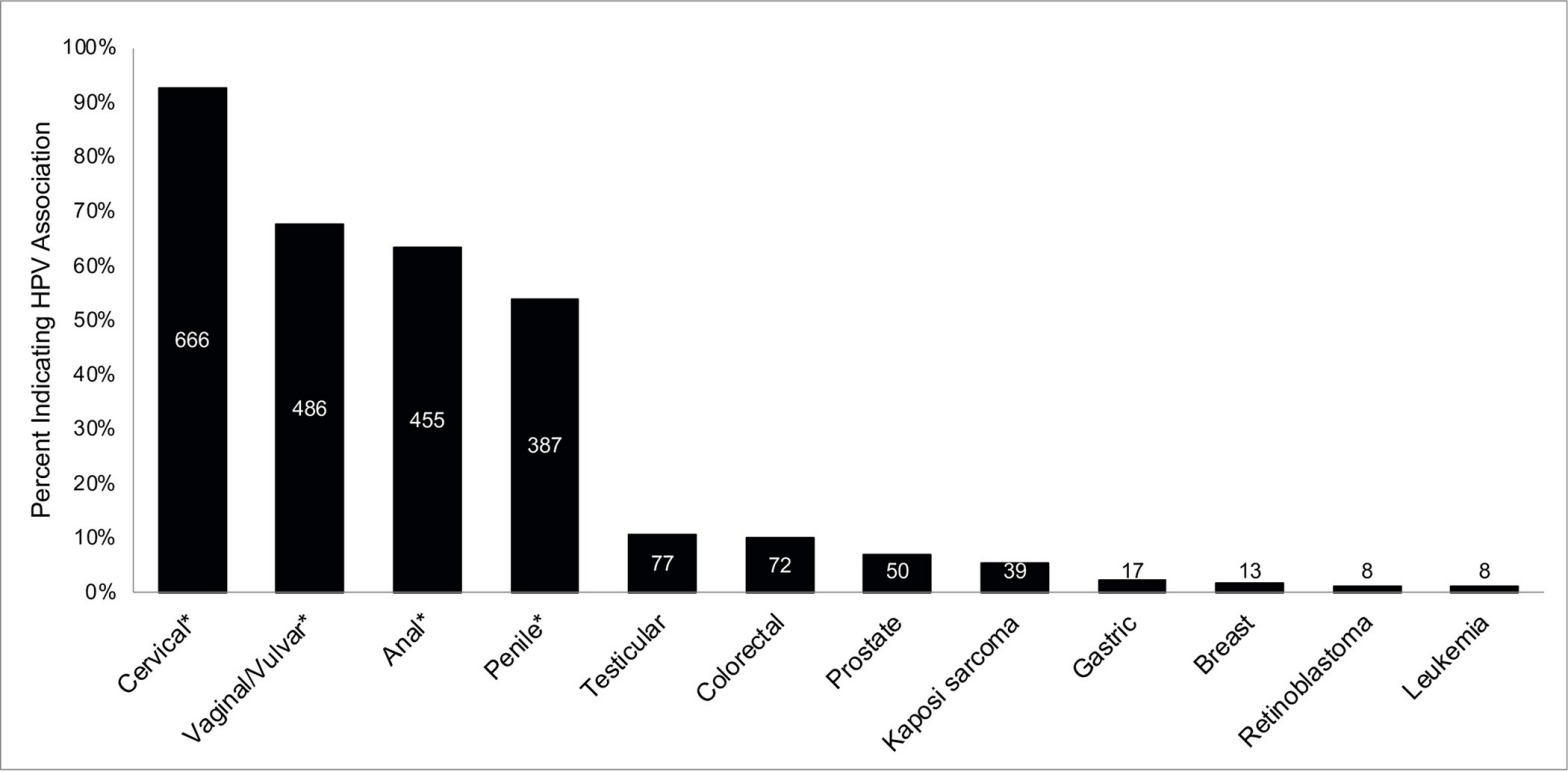
Specific cancer associations with HPV. Percentage of respondents indicating an association between HPV and each cancer type. Those indicated with an asterisk have a well-documented relationship with HPV. Labels for each bar indicate the number of participants indicating a relationship between HPV and the listed malignancy.

Participants were asked to indicate which cancers HPV could cause from a provided list of 12 different cancers- the correct answers were cervical, vaginal/vulvar, anal, and penile cancers (Fig 1). We found that 92.8% of participants correctly indicated that HPV could be a causative etiology for cervical cancer (*n* = 666). However, we found that lower percentages of participants correctly indicated HPV could be causative for vaginal/vulvar cancer (67.7%, *n* = 486), anal cancer (63.4%, *n* = 455), and penile cancer (53.9%, *n* = 387). We found that 10.7% (*n* = 77) of participants indicated HPV was a causative etiology for testicular cancer and 10.0% (*n* = 72) indicated this for colorectal cancer, both of which were incorrect.

### Multiple choice items

A greater percentage of students in the clinical-phase of training correctly answered six of the eight multiple choice items (Table 2). Specifically, greater percentages of clinical-phase students correctly identified the age at which patients are no longer indicated to receive the HPV vaccine (>45 years; 65.2% vs. 55.6%, χ^2^[1, *N* = 718] = 7.05, *p* = .008), that three HPV vaccine doses were needed to confer protection for a 16 year old patient (88.3% vs. 67.9%, χ^2^[1, *N* = 718] = 54.6, *p* < .001), and that a patient should also be considered for the Hepatitis A vaccine (84.9% vs. 78.6%, χ^2^[1, *N* = 718] = 4.54, *p* = .03) in addition to the HPV vaccine.

**Table 2:** Individual item analyses.

	Overall Correct		Pre-Clinical		Clinical		*p*
	*n*	%	*n*	%	*n*	%	
According to current recommendations, what is the earliest age patients may begin receiving the HPV vaccine?	237	33.0	118	30.1	119	36.6	.06
According to current recommendations, after what age are patients no longer indicated to receive the HPV vaccine?	430	59.9	218	55.6	212	65.2	**.01**
A woman who receives the HPV vaccine does not require cervical Pap smears (T/F)	699	97.4	378	96.4	321	98.8	**.03**
The HPV vaccine protects against all strains of the HPV virus (T/F)	679	94.6	359	91.3	320	98.5	**< .001**
Before receiving the HPV vaccine, patients should have HPV serology testing done (T/F)	540	75.2	253	64.4	287	88.3	**< .001**
Which aspect of a patient’s presentation determines the number of HPV vaccine doses needed to confer protection?	552	76.9	266	67.9	286	88.3	**< .001**
How many doses of the HPV vaccine will be necessary for a 16 year old patient?	345	48.1	189	48.2	156	48.0	.98
Assuming a patient has not received any of the vaccinations listed below, which one could be considered for the patient?	583	81.2	308	78.6	275	84.9	**.03**

In addition, a greater percentage of clinical-phase students correctly responded that women who have received the HPV vaccine still require pap-smears (98.8% vs. 96.4%, χ^2^[1, *N* = 718] = 4.62, *p* = .03), that the HPV vaccine does not protect against all strains of HPV (98.5% vs. 91.3%, χ^2^[1, *N* = 718] = 17.5, *p* < .001), and that HPV serology is not required prior to receiving the HPV vaccine (88.3% vs. 64.4%, χ^2^[1, *N* = 718] = 41.3, *p* < .001).

### Knowledge comparisons

In the univariate ANOVA analyses (Table 3) we found that knowledge of HPV differed based on a participant’s year in training (*F*[3,714] = 32.6, *p* < .001). Students in the first year of training reported the lowest knowledge percentage compared to all other years (all *p* < .001; *M* = 74.3% [72.6–76.0]) as compared to those in the second (79.4% [77.6–81.3]), third (84.2% [82.6–85.7]) and fourth years (*M* = 84.6% [82.9–86.2]) of training). We also found that knowledge of the HPV vaccine differed based on the participants’ gender identity (*F*[2,717] = 3.61, *p* = .03). Cisgender women (*M* = 81.8% [80.0–82.2]) reported higher knowledge of the HPV vaccine compared to cisgender men (*M* = 78.7% [77.1–80.2], *p* = .03). In the bivariate comparison of knowledge between allopathic (*M* = 81.3% [80.1–82.5]) and osteopathic (*M* = 79.0% [77.6–80.4]) medical students, we found that allopathic students reported a higher average knowledge score (*t*[716] = 2.53, *p* = .01).

**Table 3:** HPV knowledge assessment scores.

	Unadjusted		Adjusted	
**Year of Training**	Mean (95%CI)	*p*	Mean (95%CI)	*p*
1^st^	74.3% (72.6–76.0)	*Ref*.	76.0% (72.5–79.5)	*Ref*.
2^nd^	79.4% (77.6–81.3)	**< .001**	81.6% (78.0–85.1)	**< .001**
3^rd^	84.2% (82.6–85.7)	**< .001**	85.9% (82.2–89.6)	**< .001**
4^th^+	84.6% (82.9–86.2)	**< .001**	86.3% (82.7–89.8)	**< .001**
**Sexual Orientation**				
Heterosexual (straight)	79.8% (78.9–80.8)	*Ref*.	81.0% (77.5–84.5)	*Ref*.
Homosexual (gay/lesbian)	83.4% (79.3–87.4)	.46	83.9% (79.5–88.3)	.80
Bisexual	82.3% (79.3–85.2)	.99	82.5% (77.9–87.1)	.99
Other Sexual Orientation	80.8% (74.1–87.5)	.99	82.4% (76.8–88.0)	.99
**Gender Identity**				
Man (cisgender male)	78.7% (77.1–80.2)	*Ref*.	79.8% (77.6–82.0)	*Ref*.
Woman (cisgender female)	81.1% (80.0–82.2)	**.03**	82.8% (80.7–84.8)	**.004**
Other Gender Identity	84.2% (75.8–92.6)	.82	84.7% (75.3–94.1)	.98
**Region**				
South	82.0% (78.7–85.3)	*Ref*.	82.7% (78.4–87.0)	*Ref*.
Northeast	80.4% (78.3–82.4)	.99	82.8% (79.2–86.3)	.99
West	78.6% (76.6–80.6)	.34	81.3% (77.6–84.9)	.99
Midwest	80.6% (79.4–81.8)	.99	83.0% (79.6–86.4)	.99
**Type of Training**				
Medicine (allopathic-MD)	81.3% (80.1–82.5)	*Ref*.	83.0% (79.7–86.3)	*Ref*.
Medicine (osteopathic-DO)	79.0% (77.6–80.4)	**.01**	81.9% (78.4–85.4)	.26

In the adjusted analyses, the effect of participants’ year in training was maintained (*F*[3,713] = 32.4, *p* < .001). First-year students reported lower knowledge compared to all other years (all *p* < .001). The effect of participant gender identity was also maintained (*F*[2,713] = 5.57, *p* < .001) with cisgender women reporting higher knowledge compared to cisgender men. No additional effects of demographic or training characteristics were identified on HPV knowledge (Table 3).

## Discussion

HPV-associated cancers continue to cause mortality and morbidity in the U.S. despite wide availability of effective prevention with the HPV vaccine. Clinicians play a key role in ensuring that HPV vaccination reaches all eligible patients [14]. Medical education about the HPV vaccine is crucial so medical students can identify vaccine candidates, educate their patients about the risks and benefits, and respond to patient questions. To the best of our knowledge, this is the first multi-regional and multi-institutional study of both allopathic and osteopathic medical students’ knowledge of HPV vaccination guidelines. The current findings may be used to improve medical education about HPV-associated cancers and HPV vaccination.

Most participants (92.8%) correctly identified the connection between HPV and cervical cancer. However, smaller percentages correctly identified the link between HPV and vaginal/vulvar, anal, and penile cancers, echoing previous work [8,9]. Importantly, this previous study grouped penile and vaginal/vulvar cancer as genital cancers whereas we separated these two diagnoses. In our study, two-thirds of students linked HPV to vaginal/vulvar cancers, whereas just over half linked HPV to penile cancers. This suggests difficulty identifying HPV-related cancers in the male genital tract that both reflects the predominant societal narrative that so closely links HPV to women’s health and suggests need for concentrated educational efforts that focus specifically on HPV and anal/penile cancers [15,16].

The burden of HPV infection and HPV-related disease among men who have sex with men (MSM) remains disproportionate, and recent work has found that HPV vaccination was more common among MSM who were using HIV PrEP compared to those who were not [17]. While this is encouraging, it suggests there is a significant need to broadly improve HPV vaccination among all patients who are at risk, not just those who are already engage in preventive sexual healthcare. Research conducted among heterosexual patients found only 11.5% had received one dose of the HPV vaccination, underscoring the importance of education to broadly improve uptake of the HPV vaccine [18].

Broadly, knowledge of HPV vaccination guidelines improved with increasing years of training as second, third, and fourth-year medical students performed better than first-year students. This trend in increasing knowledge is intuitive given the progressive concentration on clinical medicine as training progresses, and these results are consistent with those from prior studies [8,10]. We found that the only questions without difference between year of training were those answered incorrectly by most students in either group. Specifically, we found that fewer than half (33.0%) of the students in this study, even those in the clinical years of training (36.6%), correctly identified patients could begin receiving the HPV vaccine at age 9. Additionally, only 48.1% of participants, including 48.0% of those in clinical training, were correct in identifying three HPV vaccine doses conferred full protection for a 16-year-old patient. The persistence of incorrect knowledge in this later phase of training suggests the need for additional training about eligibility for the HPV vaccine.

These knowledge gaps thus present an opportunity for educational intervention [19–21]. There is a demonstrable benefit that increased training among first- and second-year medical students can improve comfort and knowledge in counseling on HPV vaccination [22,23]. A follow-up study to the workshop curricula that was implemented by Evans et al. demonstrated that a year and a half later, these students retained their knowledge and continued to better advocate for the vaccine than their peers who did not participate in the workshop, and these students were also able to integrate their knowledge into increasing HPV vaccination rates in student-run free clinics [22,24].

We found several interesting demographic trends. Greater HPV knowledge was demonstrated among cisgender women as compared to cisgender men, without difference based on sexual orientation, region, or type of training. Women remain more likely to have received the HPV vaccine, and this first-hand experience is related to their greater current knowledge and future discussions of the vaccine with patients [11]. To standardize provider knowledge and to ensure that all students possess a similar foundation of knowledge related to HPV vaccination, school-wide initiatives are necessary. Even a single lecture by an expert can improve attitudes with HPV vaccination, especially in students who themselves did not have it [24–26]. Other curricula have had similar success, including a role-playing workshop conducted with 28 medical students and residents that showed improved participant knowledge, comfort, and confidence discussing the HPV vaccine [21,27,28].

### Limitations

The results of the present study should be interpreted in the context of several limitations which invite future study. First, there are slight differences in terms of the demographic composition of the study sample and those of allopathic and osteopathic medical students nationally. Relatedly, our sample is also over-representative of students attending medical school in the Midwest. For this reason, we included region as a covariate in the ANCOVA to control for any variance introduced by this variable, and its effect was not statistically significant. Second, our HPV knowledge assessment was developed specifically for this study and has not been validated or linked to clinical practice outcomes. However, the instrument was developed based on current guidelines and reviewed for accuracy by an infectious disease physician. Given that the purpose of the present study was to identify knowledge gaps to guide future educational interventions, this type of knowledge assessment was appropriate. However, we did not include oropharyngeal cancers as one of the options in our assessment of knowledge of the association of cancer types with HPV. Recent work has found knowledge gaps among medical students with respect to the association of HPV and oropharyngeal cancers [9]. This is an important area for future study to ensure that medical education curricula present comprehensive training on the malignancies associated with HPV.

Third, our survey did not assess students’ personal HPV vaccination status, which may introduce respondent bias but could also represent an avenue of future inquiry into HPV vaccination knowledge and practice patterns. Future study is also needed to directly link knowledge deficits to clinical decision-making regarding HPV vaccination as well as to develop common standards for medical education about HPV vaccination. Finally, we also acknowledge the limitation of studying medical students given their inability to practice independently. However, studying this population is essential for curriculum design, improvement, and preparation for practice. Additional work is needed to determine whether the pattern of findings identified in this study are also present among practicing physicians.

## Conclusion

Increased knowledge about HPV-related cancers and HPV vaccination as well as confidence discussing the HPV vaccine can positively impact its uptake and continue to decrease the prevalence of HPV-related cancers. This begins with giving medical students a strong foundation on the effects of HPV infection, the benefits of vaccination, and when/to whom it should be administered. While many studies have focused on the general knowledge base of medical students in specific regions and at specific institutions, we demonstrate multi-regional osteopathic and allopathic knowledge of HPV vaccination rationale and guidelines, highlighting the gaps in knowledge where educational interventions are needed. We found deficits in medical students’ ability to link HPV to vaginal, vulvar, anal, and especially penile cancers, including markedly decreased knowledge in the pre-clinical years. The focus of future work should now shift to the development and implementation of curricular interventions. Students could greatly benefit from lectures and workshops dedicated specifically to non-cervical HPV-related cancers and how to discuss the HPV vaccine with patients. With a new cohort of physicians prepared to address HPV vaccination, we may more successfully decrease the prevalence of deaths from cancers preventable with the HPV vaccine.

## Supporting information

S1 Table Allopathic and osteopathic sample student demographics. Comparison of sample demographics of allopathic and osteopathic medical student populations in this study.
(DOCX)

S2 Table Study and national demographics. Comparison of sample demographics to national allopathic and osteopathic medical student populations.
(DOCX)

S1 File Study instrument. This file contains the study instrument that was used to collect data for this study.
(PDF)

S2 File Data file. This file contains the raw data collected in this study.
(SAV)
