# Supplementary material for: Knowledge of human papillomavirus vaccination: A multi-institution, cross-sectional study of allopathic and osteopathic medical students
Source: PLoS One. 2023 Jan 11;18(1):e0280287. doi: 10.1371/journal.pone.0280287 (PMC9833510; doi:10.1371/journal.pone.0280287)
Supplement: S1 Table — Comparison of sample demographics of allopathic and osteopathic medical student populations in this study. (DOCX) [file pone.0280287.s001.docx]

**Supplemental Table 1.** Allopathic and Osteopathic Sample Student Demographics.

|  | Medicine (allopathic-MD) | | Medicine (osteopathic-DO) | |
| --- | --- | --- | --- | --- |
| **Year of Training** | ***n*** | **%** | ***n*** | **%** |
| 1st year | 106 | 27.7% | 98 | 29.2% |
| 2nd year | 83 | 21.7% | 106 | 31.5% |
| 3rd year | 88 | 23.0% | 69 | 20.5% |
| 4th year | 105 | 27.5% | 63 | 18.8% |
| **Race/Ethnicity** |  |  |  |  |
| White | 233 | 61.0% | 183 | 54.5% |
| Black | 13 | 3.40% | 14 | 4.20% |
| Hispanic/Latino | 24 | 6.30% | 23 | 6.80% |
| Asian | 112 | 29.3% | 127 | 37.8% |
| Other Race | 15 | 3.90% | 11 | 3.30% |
| **Sexual Orientation** |  |  |  |  |
| Heterosexual (straight) | 310 | 81.4% | 294 | 87.8% |
| Gay/Lesbian | 24 | 6.30% | 16 | 4.80% |
| Bisexual | 33 | 8.70% | 20 | 6.00% |
| Other Sexual Orientation | 14 | 3.70% | 5 | 1.50% |
| **Gender Identity** |  |  |  |  |
| Man | 137 | 35.9% | 125 | 37.2% |
| Woman | 240 | 62.8% | 210 | 62.5% |
| Other Gender Identity | 5 | 1.30% | 1 | 0.30% |
| **Region** |  |  |  |  |
| South | 61 | 16.0% | 7 | 2.10% |
| Northeast | 61 | 16.0% | 77 | 23.0% |
| West | 32 | 8.40% | 117 | 34.9% |
| Midwest | 227 | 59.6% | 134 | 40.0% |

Comparison of sample demographics of allopathic and osteopathic medical student populations in this study.
