# Supplementary material for: Knowledge of human papillomavirus vaccination: A multi-institution, cross-sectional study of allopathic and osteopathic medical students
Source: PLoS One. 2023 Jan 11;18(1):e0280287. doi: 10.1371/journal.pone.0280287 (PMC9833510; doi:10.1371/journal.pone.0280287)
Supplement: S2 Table — Comparison of sample demographics to national allopathic and osteopathic medical student populations. (DOCX) [file pone.0280287.s002.docx]

**Supplemental Table 2**. Study and national demographics.

|  | Medicine (allopathic-MD)^a^ | | | | Medicine (osteopathic-DO)^b^ | | | |
| --- | --- | --- | --- | --- | --- | --- | --- | --- |
|  | National Data | | Sample Data | | National Data | | Sample Data | |
| **Race** | *n* | % | *n* | % | *n* | % | *n* | % |
| African-American or Black | 8,444 | 8.3% | 13 | 3.40% | 1,034 | 3.3% | 14 | 4.20% |
| Caucasian or White | 53,227 | 52.6% | 233 | 61.0% | 18,156 | 58.1% | 183 | 54.5% |
| Hispanic or Latino/a/x | 9,221 | 9.1% | 24 | 6.30% | 1,883 | 6.0% | 23 | 6.80% |
| American Indian or Alaska Native | 1,331 | 1.3% | 112 | 29.3% | 117 | 0.4% | 127 | 37.8% |
| Asian | 24,654 | 24.4% | 15 | 3.90% | 7,337 | 23.5% | 11 | 3.30% |
| Other | 4,310 | 4.3% |  |  | 2,701 | 8.6% |  |  |
| **Gender Identity** |  |  |  |  |  |  |  |  |
| Man | 44,906 | 48.5% | 137 | 35.9% | 16,562 | 52.3% | 125 | 37.2% |
| Woman | 47,726 | 51.5% | 240 | 62.8% | 15,088 | 47.7% | 210 | 62.5% |
| Other (Unspecified) | - | - | 5 | 1.30% | 13 | 0.04% | 1 | 0.30% |
| **Phase of Training** |  |  |  |  |  |  |  |  |
| 1st year | - | - | 106 | 27.7% | 8,805 | 27.8% | 98 | 29.2% |
| 2nd year | - | - | 83 | 21.7% | 8,094 | 25.6% | 106 | 31.5% |
| 3rd year | - | - | 88 | 23.0% | 7,685 | 24.3% | 69 | 20.5% |
| 4th year | - | - | 105 | 27.5% | 7,079 | 22.4% | 63 | 18.8% |

Comparison of sample demographics to national allopathic and osteopathic medical student populations.

1. Association of American Medical Colleges. *2020 Facts: Enrollment, Graduates, and MD/PhD Data.* Available from: <https://www.aamc.org/data-reports/students-residents/interactive-data/2020-facts-enrollment-graduates-and-md-phd-data>
2. American Association of Colleges of Osteopathic Medicine. *AACOM Reports on Student Enrollment.* Available from: https://www.aacom.org/reports-programs-initiatives/aacom-reports/student-enrollment
