# Supplementary material for: Knowledge of human papillomavirus vaccination: A multi-institution, cross-sectional study of allopathic and osteopathic medical students
Source: PLoS One. 2023 Jan 11;18(1):e0280287. doi: 10.1371/journal.pone.0280287 (PMC9833510; doi:10.1371/journal.pone.0280287)
Supplement: S1 File — This file contains the study instrument that was used to collect data for this study. (PDF) [file pone.0280287.s003.pdf]

RECRUITMENT MATERIALS

Initial message:

Dear Medical Student,

You are invited to participate in an educational research study about preventive medicine among various patient groups. You will be asked to review a patient vignette and answer follow up questions. The results will be used to improve clinical vignettes in medical education. You will have the option of receiving a debrief message following the completion of our study.

Immediately after completing the study, **you will receive a $10 gift card** as compensation for your time. We estimate participating in this study will take approximately 20 minutes. All responses will be **completely anonymous and confidential**, so please answer honestly.

CLICK THIS LINK to begin the study:
[*LINK INSERTED HERE*]

This study is being conducted by Samuel Bunting (MS4) and Sarah Garber, PhD, of the Chicago Medical School, and College of Pharmacy, respectively at Rosalind Franklin University in North Chicago, Illinois, USA. If you have questions or concerns about this survey, please contact Sarah Garber at sarah.garber@rosalindfranklin.edu or Samuel Bunting at samuel.bunting@my.rfums.org.

This study was approved by the Institutional Review Board of Rosalind Franklin University (Protocol: COP-20-256 on September 24, 2020).

Follow up message once a participant indicated interest:

Dear Medical Student,

Thank you for signing up to participate in this study related to preventive medicine in various patient populations! Please click the link below or copy and paste the URL below to be taken to the study portal to begin the study. Once you have finished the study, you will receive the gift card redemption instructions via email.

As a reminder, this study must be completed on a device with a physical keyboard. If you are not utilizing a device with a physical keyboard now, please come back to this study when you

are using a device with a physical keyboard.

Follow this link to the Study Portal: [*LINK INSERTED HERE*]

Part 1: INFORMED CONSENT FOR RESEARCH PARTICIPATION

Evaluating Students' Perceptions of Preventive Medicine in Various Patient Groups

You are invited to be in a research study of students' perceptions of Preventive Medicine in Various Patient Groups. You were selected as a possible participant because you are a student in a health professions educational program in the United States. We ask that you read this form and ask any questions you may have before agreeing to be in the study.

This study is being conducted by: Samuel Bunting, MS4, Chicago Medical School, Rosalind Franklin University of Medicine and Science and Sarah Garber, PhD, College of Pharmacy, Rosalind Franklin University of Medicine and Science.

Procedures: If you agree to be in this study, we would ask you to do the following things:

- Complete a study instrument regarding about preventive medicine among various patient groups.
- The survey will take approximately 20 minutes and you will be asked to complete it once.

Confidentiality: The records of this study will be kept private. In any sort of report we might publish, we will not include any information that will make it possible to identify a subject. Research records will be stored securely and only researchers will have access to the records. No information that you submit as a part of the research activities or for the administration of the study completion incentive will be utilized to identify a specific respondent's responses. As data required for administration of the gift card incentive following cessation of the study is entered and maintained by Qualtrics, no member of the research team will have access to this information. Confidentiality will be maintained pursuant to the data protection policies of Qualtrics and any interface the researchers have with this data will be protected by the encryption and security of the RFUMS network.

Voluntary Nature of the Study: Participation in this study is voluntary. Your decision whether or not to participate will not affect your current or future relations with Rosalind Franklin

University. If you decide to participate, you are free to not answer any question or to withdraw at any time.

Contacts and Questions: Please ask any questions you have now. If you have questions later, you may contact Samuel Bunting at Samuel.bunting@my.rfums.org, or Sarah Garber, PhD at sarah.garber@rosalindfranklin.edu. If you have any questions or concerns regarding your rights as a subject in this study, you may call the IRB office (phone: 847-578-8713 or email: IRB@rosalindfranklin.edu).

You may print a copy of this information to keep for your records.

This research study COP-20-256 received approval as an EXEMPT human subjects research project from the Rosalind Franklin University IRB on September 24, 2020.

Statement of Consent: I have read the above information and have received answers to any questions I asked. I consent to take part in the study.

◯ YES, I consent to participation.

◯ NO, I do NOT consent to participation.

Are you over 18 years of age?

◯ Yes

◯ No

Part 2: HPV KNOWLEDGE ASSESSMENT

Thank you for agreeing to participate in this research study! We appreciate your time.

We will present a series of items regarding knowledge of the human papillomavirus (HPV). Please answer to the best of your ability.

Which of the following cancers does the HPV vaccine protect against? *Select all that apply*

- Cervical*
- Anal*
- Penile*
- Vaginal/Vulvar*
- Colorectal
- Gastric
- Retinoblastoma
- Breast
- Prostate
- Testicular
- Leukemia
- Kaposi sarcoma

Which aspect of a patient's presentation determines the number of HPV vaccine doses needed to confer protection?

- Patient age at first vaccination*
- Patient immunocompromised status
- Results of HPV serology
- Family history of HPV-related cancers

How many doses of the HPV vaccine will be necessary for a 16 year old patient?

- 1
- 2
- 3*
- 4

According to current recommendations, what is the *earliest* age patients may begin receiving the HPV vaccine?

- 1 year
- 3 years
- 6 years
- 9 years*
- 11 years
- 15 years

According to current recommendations, after what age are patients no longer indicated to receive the HPV vaccine?

- After age 45*
- After age 16
- After age 75
- After age 21

A woman who receives the HPV vaccine does not require cervical Pap smears (T/F)

- TRUE
- FALSE*

The HPV vaccine protects against all strains of the HPV virus (T/F)

- TRUE
- FALSE*

Before receiving the HPV vaccine, patients should have HPV serology testing done (T/F)

- TRUE
- FALSE*

Assuming the patient has not received any of the vaccinations listed below, which one could be considered for a patient receiving the HPV vaccine?

- Hepatitis A vaccine*
- Tuberculosis vaccine
- Yellow fever vaccine
- Hepatitis D vaccine

*CORRECT answers are indicated with asterisks.

Part 3: DEMOGRAPHICS

The final group of items will ask some general demographic information.

1. Which academic program are you enrolled in?

○ Medicine (allopathic-MD)

○ Medicine (osteopathic-DO)

2. What is your academic standing in your program?

○ 1st year

○ 2nd year

○ 3rd year

○ 4th year

○ Other ____________________

3. Please indicate your age in years: ____________________

4. Which of the following was your sex assigned at birth?

○ Male

○ Female

5. What is your gender identity?

○ Man (cisgender male)

○ Woman (cisgender female)

○ Transgender man

○ Transgender woman

○ Gender nonbinary

○ Other ____________________

6. What is your race/ethnicity?

○ African-American (Black)

○ Caucasian (White)

○ Hispanic/Latino

○ Native American

○ Asian

○ Other ____________________

7. Which of the following best describes your sexual orientation?

○ Heterosexual (straight)

○ Homosexual (gay)

○ Bisexual

○ Other ____________________

8. In which state do you attend school?

DROP DOWN MENU OF ALL 50 U.S. STATES
